# Sewage surveillance for assessing clinical antibiotic resistance prevalence: Combining metagenomic and phenotypic data

**DOI:** 10.1016/j.onehlt.2026.101485

**Published:** 2026-06-18

**Authors:** Carl-Fredrik Flach, Fanny Berglund, Gilbert Osena, Patricia M.C. Huijbers, D.G. Joakim Larsson

**Affiliations:** a Centre for Antibiotic Resistance Research in Gothenburg (CARe), University of Gothenburg, Gothenburg, Sweden; b Institute of Biomedicine, Department of Infectious Diseases, University of Gothenburg, Gothenburg, Sweden

**Keywords:** Wastewater, *Escherichia coli*, Metagenomics, Antibiotic resistance gene, Resistance rate, Clinical isolates

## Abstract

Surveillance of antibiotic resistance in clinical isolates is a cornerstone for the management of bacterial infections but is limited in large parts of the world, often due to lack of resources. Sewage surveillance has been proposed as a promising, resource-efficient complement to the traditional surveillance approach based on samples from many individual patients. Both phenotypic data on resistance in sewage isolates and abundance of antibiotic resistance genes in sewage have been shown to correlate with resistance prevalence in clinical isolates. Here, we aimed to directly compare and combine an isolate-based and a gene-based sewage surveillance approach to evaluate what best can reflect clinical resistance rates. The two approaches, based on susceptibility testing of collected *E. coli* isolates and metagenomic sequencing, respectively, were applied to municipal sewage samples collected in ten European countries. The data generated was related to available data on resistance to aminopenicillins, fluoroquinolones, third generation cephalosporins and aminoglycosides prevalence in clinical *E. coli* isolates using beta regression models. None of the tested individual predictors were superior across all four investigated classes of antibiotics. For modelling of aminopenicillin resistance, a clearly higher R^2^ value was obtained when isolate-based and gene-based data was combined as predictors, also after adjusting for the number of included variables. We conclude that there could be a value of including both isolate- and gene-based sewage data for predictions of resistance rates in clinical isolates, while emphasizing the value of linking predictors to specific species and classes of antibiotics.

## Highlights

• Isolate- and gene-based sewage surveillance approaches were compared and combined.
• Sewage data was used to model resistance prevalence in clinical isolates.
• No sewage predictor was superior across all tested antibiotic classes.
• Combining isolate- and gene-based data clearly improved model fit in some cases.

## Graphical abstract

**Figure:**
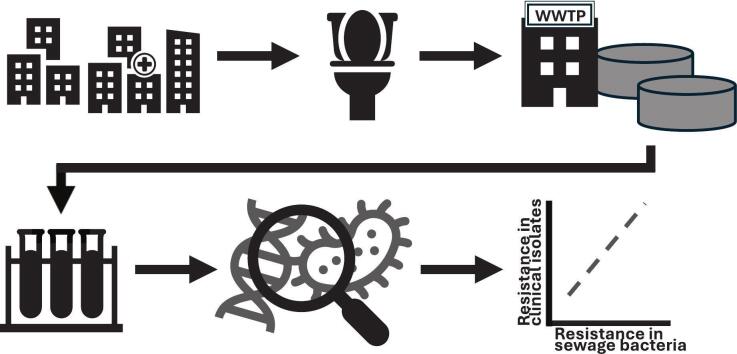

## 1 Introduction

The overwhelming majority of all antibiotic treatments are empirical, meaning that the antibiotic susceptibility of the infectious agent to be treated is not known. Hence, it is invaluable that treatment guidelines are based on up-to-date regional surveillance data, which inform about the prevalence of resistance to different types of antibiotics in various bacterial pathogens. A prerequisite for generation of representative surveillance data is the collection and analysis of bacterial isolates from many individuals. This is a resource-demanding process, which often limits the collection of data, especially in many low- and middle-income countries [1], [2].

Wastewater-based epidemiology refers to the use of wastewater analyses to provide information about different health-related variables in human populations. An example is polio, which has been monitored for decades in municipal sewage [3]. During the SARS-CoV-2 pandemic, the potential of a wider use of sewage epidemiology became apparent [4]. While sewage monitoring for human viruses is already in place in many regions of the world, the potential to generate population-level surveillance data on antibiotic resistance in a similar manner has more recently received increased attention [5], [6], [7], [8], [9]. Given that a single sewage sample can contain shed bacteria from thousands of individuals, it could become an attractive, resource-efficient complement to the classical surveillance approach based on compiled diagnostics data from many individual patients.

In both a local Swedish study and a study of ten European countries, we have shown correlations between resistance rates in clinical and sewage *Escherichia coli* isolates [10], [11]. These studies demonstrated that sewage isolates could inform whether resistance to one antibiotic is more common than resistance to another in clinical isolates [11], and that the relationship between resistance prevalence in sewage and clinical isolates could remain relatively stable across countries [10]. In addition, metagenomic analyses of complex sewage samples have emerged as an alternative approach [12], [13], [14], [15]. Both Pärnänen et al. [15] and Karkman et al. [13] showed associations between the abundance of antibiotic resistance genes (ARGs) in sewage and the prevalence of resistance in clinical isolates from the corresponding countries. However, in the study by Karkman et al. the best predictor found for clinical resistance rates was not the total abundance of ARGs or the abundance of certain classes of ARGs in the sewage, but rather the abundance of the *intI1* gene. This gene encodes the integrase of class 1 integrons, which often is associated with antibiotic resistance in, for example, *E. coli* [16], [17]. Results from several environmental studies have accordingly suggested *intI1* as a proxy for antibiotic resistance [18], [19]. Given that *intI1* is not specifically associated with any particular class of antibiotics, nor specific to an individual bacterial species, it is a general resistance marker, similarly to *e.g.* total ARGs.

Although both isolate-based and gene-based sewage analyses have shown some promise in predicting the prevalence of resistance in regional clinical isolates, a direct comparison between the two sewage surveillance approaches is still missing. Nor has any study explored the value of combining them. Here, we aimed to compare and combine the two approaches to evaluate what type of sewage data that best reflect clinical resistance rates. To achieve this, previously generated phenotypic isolate-based data [10] was complemented with metagenomic DNA sequencing data for municipal sewage samples collected in ten European countries. Subsequently, relationships between publicly reported antibiotic resistance prevalence in clinical *E. coli* and sewage variables were investigated using beta regression models.

## 2 Materials and methods

### 2.1 Sewage samples and *E. coli* isolates

This work relied on sewage samples described in a previous study [10]. Briefly, the samples were collected as composite 24 h samples from the influents to ten major municipal wastewater treatment plants located in ten European countries from December 2016 to December 2017 [10]. In the previous study, approximately 250 *E. coli* isolates from each sample were screened against a panel of antibiotics including ampicillin, cefotaxime, ceftazidime, ciprofloxacin and gentamicin. The screened antibiotics represented antibiotic classes covered in the reports from the European Antibiotic Resistance Surveillance Network (EARS-Net) compiling resistance data from clinical invasive *E. coli* isolates across Europe. Depending on when the sewage sample was collected, resistance data from either the 2016 or the 2017 EARS-Net report [20], [21] were used to match the sewage resistance data as closely as possible in time (Supplementary Table S1). The phenotypic resistance data for the sewage *E. coli* has been described in detail before and was also included in this study [10]. Using the data for the sewage isolates, a multiple antibiotic resistance index (MARI) [22] was calculated for each country as the mean MARI across the isolates using the following formula; (aggregated number of resistance calls for all isolates) / (number of antibiotic classes tested × number of isolates tested). Resistance against cefotaxime and/or ceftazidime in an isolate was counted as one resistance call as both antibiotics are third-generation cephalosporins.

### 2.2 DNA isolation from sewage samples and metagenomic sequencing

On the same day as being collected, 20 mL aliquots of the composite sewage samples were passed through 0.45 pore size filters, which were subsequently stored at −20 °C until all samples could be further processed in parallel. Total DNA was isolated from the filters using the DNeasy PowerWater Kit (Qiagen, Hilden, Germany) according to the manufacturer's instructions. The DNA concentration was measured using a Qubit® 2.0 instrument (Life technologies, Carlsbad, CA, USA) and stored at −20 °C until further analysis.

DNA was shipped to Science for Life laboratories' National Genomics Infrastructure (Solna, Sweden) for metagenomic sequencing. Sequencing libraries were prepared using Illumina DNA PCR-free Prep (Illumina, San Diego, CA, USA). The samples were multiplexed and sequenced on 1.75 lanes of NovaSeq 6000 S4 flow cells using paired-end sequencing (2 × 150 bp).

### 2.3 Bioinformatics processing

The sequencing data was quality controlled and adapter filtered using bbduk from the bbmap suite (version 38.96) with the parameters “ktrim=r ref=adapters tpe tbo trimq=20 minlen=50 qtrim=rl” [23]. The quality controlled data was mapped against the human reference genome hg38 using Bowtie2 (version 2.4.5) [24] and all fragments with a significant match were removed from the dataset. To predict 16S rRNA sequences, the data was analyzed using seqtk (version 1.3) and metaxa2 (version 2.2.3) with the parameters “seqtk seq -a < input.fastq> | metaxa2_x “[25], [26]. The data was then searched for mobile antibiotic resistance genes using Diamond (version 2.0.15) against the ResFinder database (downloaded June 2022) with parameters “blastx -max-target-seqs 0 –id 70 –query-cover 60″ [27], [28]. The results from the alignment against ResFinder were parsed so that only fragments with a sequence identity match higher than 90% were kept, and those matches were converted into count tables. Based on clustering of the ResFinder database using the usearch “cluster_fast” algorithm [29] with an amino acid identity threshold of 90%, a resistance gene category called genes_clustered_at_90 was created. The count table was then aggregated into the categories resistance_gene_class, genes_clustered_at_90 and gene. Mobile genetic elements, including *intI1,* were identified by aligning the data to MobileGeneticElementDatabase [30] using diamond blastx and converted into count tables with the same parameters as above.

The *E. coli* genomes were downloaded using GEnView with parameters “—taxa *Escherichia coli*” [31], by parsing the NCBI assembly_summary file, only unique strains were filtered out and kept. The unique *E. coli* genomes were searched for antibiotic resistance genes using Diamond against the ResFinder database with the parameters “blastx –subject-cover 70 –id 70 –max-target-seqs 0”. The results were parsed using an in-house python script and only matches with an identity above 90% were kept. The resistance gene counts were then summarized and ranked by the category “gene_clustered_at_90”. Using the ranked list of resistance genes present in *E. coli* together with phenotype information gathered from the ResFinder database, the most abundant resistance genes in *E. coli* were further divided into the categories “all”, “aminopenicillin resistance genes”, “fluoroquinolone resistance genes”, “3^rd^ generation cephalosporin resistance genes” and “aminoglycoside resistance genes” (Supplementary Table S2).

### 2.4 Data analysis

All data generated from the bioinformatic analysis as well as the phenotypic resistance data for sewage and clinical *E. coli* isolates, was loaded into R (version 4.2.2) together with metadata of the samples. The relative abundance of the ARGs in the sequencing data was calculated by first dividing the fragment count for each ARG with the length of the corresponding ARG and then by the total number of predicted 16 s rRNA fragments in each sample. The prevalence of clinical resistance was modeled with beta regression, which is a well suited for modelling proportion data, using the function *betareg* v. 3.1.4 [32] and the pseudo R^2^ values were reported. To compare performance between models, we calculated adjusted R^2^ values which account for the number of predictor variables in each model using the equation:

$$1-\frac{\left(N-1\right)}{\left(N-1-p\right)}\left(1-R^{2}\right)$$

Where N is the number of observations, p is the number of predictor variables and R^2^ the pseudo R^2^ from the beta regression.

To assess the accuracy of the models in predicting resistance rates in the clinical isolates, predictions were generated from each model using the leave-one-out cross-validation (LOOCV) method. Subsequently, the mean absolute percentage error (MAPE) was calculated from the LOOCV predictions for each model per antibiotic class. Differences between resulting residuals from multivariable models and corresponding univariable models were tested using one-tailed paired Student's *t*-tests (α = 0.05).

## 3 Results

### 3.1 Antibiotic resistance prevalence in sewage samples

The results from antibiotic susceptibility testing of *E. coli* isolates collected from the sewage samples have been described in detail before [10]. Briefly, a clear north-south gradient could be observed for ampicillin (an aminopenicillin and beta-lactam) and ciprofloxacin (a fluoroquinolone) resistance rates, where the highest rates were observed in the southern countries and the lowest in the most northern countries. For gentamicin (an aminoglycoside) and the cefotaxime/ceftazidime (third-generation cephalosporins), for which the resistance rates were generally lower, no such pattern was observed.

**Fig. 1:**
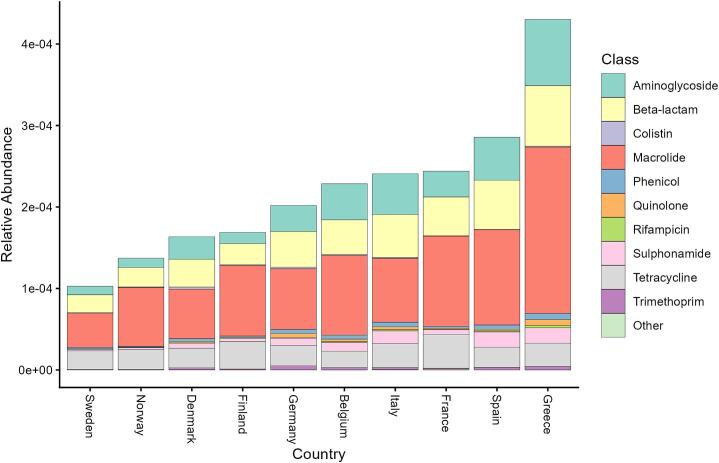
Relative abundance of ARGs in sewage samples divided between different antibiotic classes. ARG counts were normalized to 16S.

For this study, the previously generated isolate-based data was complemented with gene-based metagenomic sequencing data from the exact same samples. The number of obtained reads ranged from 519 to 1272 million per sample, and from this data the abundances of ARGs were retrieved. Throughout this study, we only considered ARGs annotated as acquired in the ResFinder database. The abundances of total ARGs in the sewage samples displayed a similar north-south gradient as described above for ampicillin and ciprofloxacin resistance rates in *E. coli*. Such a pattern was also observed for aminoglycoside and beta-lactam ARGs, but not for fluoroquinolone ARGs (Fig. 1).

### 3.2 Models based on sequencing data from sewage samples

To evaluate how well sewage data correlates with resistance prevalence in clinical *E. coli* isolates, several different models were tested. First, we investigated how the total abundance of ARGs correlated with the clinical resistance rates. The R^2^ values for the beta regression models ranged from 0.44 to 0.59 for the four investigated antibiotic classes.

When instead only including ARGs conferring resistance to the respective antibiotic class in the explanatory variable, better model fits were observed for the aminopenicillin, third-generation cephalosporin and aminoglycoside resistance models (R^2^ values of 0.69, 0.61 and 0.76, respectively), but a worse fit was found for the fluoroquinolone resistance model (R^2^ 0.37) (Table 1).

**Table 1:** Obtained R^2^ values from beta regression analysis, clinical resistance rates *vs.* sewage data.

	AP-res	FQ-res	3GC-res	AG-res	Mean^a^
**Gene-based data**					
Total ARGs	0.48	0.45	0.44	0.59	0.49
ARGs for respective antibiotic class	0.69	0.37	0.61	0.76	0.61
Top ten *E. coli* ARGs	0.60	0.69	0.55	0.77	0.65
Top ten *E. coli* ARGs for respective antibiotic class	0.70	0.37	0.75	0.77	0.65
*intI1*	0.62	0.74	0.59	0.77	0.68

**Phenotypic isolate-based data**					
Resistance rates for respective antibiotic class	0.67	0.56	0.00	0.15	0.34
MARI	0.60	0.51	0.34	0.49	0.48

**Gene-based + isolate-based data**					
*intI1* + resistance rates	0.79	0.74	0.59	0.77	0.72
*intI1* + MARI	0.71	0.75	0.58	0.77	0.70
Top ten *E. coli* ARGs + resistance rates	0.82	0.71	0.57	0.77	0.72
Top ten *E. coli* ARGs + MARI	0.73	0.74	0.57	0.80	0.71
Top ten *E. coli* ARGs for respective antibiotic class + resistance rates	0.86	0.70	0.77	0.77	0.78
Top ten *E. coli* ARGs for respective antibiotic class + MARI	0.80	0.75	0.76	0.81	0.78

ARGs: antibiotic resistance genes; AP-res: aminopenicillin resistance; FQ-res: fluoroquinolone resistance; 3GC-res: third-generation cephalosporin resistance; AG-res: aminoglycoside resistance; MARI: multiple antibiotic resistance index.

a The mean of the R^2^ values obtained from the four models.

In the previous study by Karkman et al. [13], model fits were improved by focusing on the ARGs most commonly found in *E. coli* as compared to using total ARGs. We therefore tested models that only included the top ten ARGs in *E. coli* for each class (see Supplementary Table S2 for included ARG clusters). Compared to the models including all detected ARGs for respective antibiotic class, the top ten *E. coli* models showed a better model fit for third-generation cephalosporin resistance (R^2^ 0.75), while it resulted in very similar R^2^ values for the other three antibiotic classes (Table 1). In the case of fluoroquinolone resistance, this was not surprising given that the total abundance of those top ten genes was very close to the total abundance of all resistance genes of that class, on average 97% (Supplementary Fig. 1). When models instead included the overall top ten ARGs in *E. coli*, irrespective of which antibiotic the genes confer resistance to, a clearly higher model fit was observed for the ciprofloxacin resistance model (R^2^ 0.69), but not for any of the other antibiotic classes (Table 1).

In Karkman et al., the models showing the best fits were not those including any ARGs but rather a single gene often linked to antibiotic resistance, *intI1*. Models based on *intI*1 abundance as the sole explanatory variable were therefore also tested here. The resulting models showed a higher mean model fit than any of the other tested gene-based approaches, with R^2^ values ranging from 0.59 to 0.77 for the different antibiotic classes (Table 1).

### 3.3 Models based on phenotypic resistance data from sewage isolates

We have in an earlier publication modeled clinical resistance data from the 2016 EARS-Net report by linear regression using resistance rates in sewage *E. coli* isolates for the respective antibiotic class as explanatory variable [10]. Here, beta regressions models were used to model temporally matched clinical resistance rates, which resulted in R^2^ values similar to the previously published ones: 0.67, 0.56, 0.00, and 0.15 for the aminopenicillin, fluoroquinolone, third-generation cephalosporin and aminoglycoside resistance models, respectively (Table 1). We also calculated a MARI for each country based on the phenotypic resistance data for the sewage isolates. These MARIs were tested as an explanatory variable, which like total ARGs and *intI1* is an explanatory variable not specific to any particular antibiotic class. The R^2^ values for the MARI-based models were higher for the third-generation cephalosporin (0.34) and aminoglycoside (0.49) resistance models but lower for the aminopenicillin (0.60) and fluoroquinolone (0.51) resistance models, compared to models where phenotypic data specific to the respective antibiotic class was used as the explanatory variable (Table 1).

### 3.4 Comparison of models using a single explanatory variable

When all models using a single explanatory variable were compared, no approach or individual predictor showed clearly superior model fit across all investigated classes of antibiotics (Table 1). The only case when an individual predictor resulted in clearly better model fit than all the other explanatory variables was when third generation cephalosporin resistance was modeled. The highest R^2^ value (0.75) was seen for the model including the abundance of the top ten cephalosporin resistance genes in *E. coli* as the explanatory variable, whereas the second best model fit (R^2^ = 0.59) was observed for the model including *intI1* abundance.

### 3.5 Models based on combinations of sequencing and phenotypic data

To test if better model fits could be obtained by combining isolate-based and gene-based sewage data, we first evaluated models where *intI1* abundance in sewage and resistance rates in sewage isolates were included as two explanatory variables. Compared to when the included explanatory variables were evaluated individually, the R^2^ was increased for the aminopenicillin resistance model (0.79) but not for the other models. A very similar trend was seen when top ten *E. coli* ARGs, irrespective of which antibiotic they confer resistance to, and phenotypic resistance rates were combined (Table 1).

Next, we explored combined models where neither the phenotypic nor the genetic sewage data was specific to any class of antibiotics. We did this by combining MARI with either *intI1* abundance or abundance of the ten most common *E. coli* ARGs (regardless of antibiotic class) as explanatory variables. In both cases, the mean R^2^ value was very similar to what was obtained for the former multivariable model approaches, although lower R^2^ values were observed for the aminopenicillin resistance models (Table 1).

Finally, we tested models where abundance data for the ten most common *E. coli* ARGs for respective antibiotic class was combined with isolate-based data. The class-specific gene-based data was combined with either resistance rates to individual antibiotics (meaning that only class-specific data is used in the models) or with MARI, which is not class-specific. For both these approaches R^2^ values of at least 0.7 were obtained for all four models, which was not seen for any of the other tested approaches. In line with this, they resulted in the highest mean R^2^ values of all tested approaches (Table 1). Compared to when the included explanatory variables were evaluated using univariable model approaches, these combined models clearly increased R^2^ values for the aminopenicillin and fluoroquinolone resistance models (Table 1). This was further manifested when the number of explanatory variables were considered by calculating adjusted R^2^ values (Supplementary Table S3).

When the models were evaluated through LOOCVs, the combined models including phenotypic resistance rates all resulted in a higher measured performance than the corresponding univariable models for predictions of aminopenicillin resistance. The model combining phenotypic resistance rate and abundance of the most common aminopenicillin resistance genes in *E. coli* showed the best performance (MAPE = 10.8) (Supplementary Table S4). However, when the residuals of combined and univariable models were compared, no statistically significant differences were observed. For the other antibiotic classes, univariable models showed the lowest MAPE. When fluoroquinolone resistance in clinical isolates was modeled, this was largely because models incorporating fluoroquinolone resistance in sewage isolates (*i.e.* fluoroquinolone resistance rates or MARI) performed poorly in predicting clinical resistance rates in Spain (Supplementary Fig. S1).

### 3.6 Combining data specific to different antibiotic classes

**Fig. 2:**
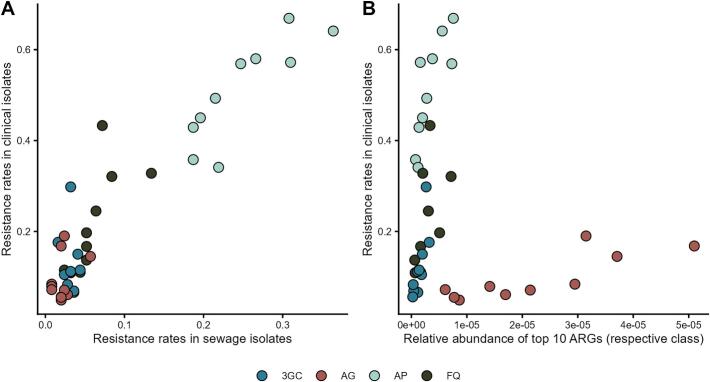
Resistance rates in clinical isolates plotted against isolate-based (A) or gene-based (B) sewage data for four different antibiotic classes: aminopenicillins (AP), fluoroquinolones (FQ), third-generation cephalosporins (3GC) and aminoglycosides (AG). Data was collected from ten different European countries. Clinical resistance rates correspond to data reported for invasive *E. coli* isolates to EARS-Net for the years 2016 and 2017. The isolate-based sewage data used is resistance rates for 250 screened *E. coli* isolates per country and antibiotic class. The gene-based sewage data is the abundance of the ten most common acquired *E. coli* ARGs per country and antibiotic class, normalized to 16S.

To visualize how well different types of sewage variables could reflect which types of resistance that are more common than other in clinical isolates, resistance rates in the clinical isolates for the four investigated antibiotic classes were plotted against the corresponding resistance rates in sewage isolates (Fig. 2A) or the abundance of the top ten *E. coli* ARGs for respective antibiotic class (Fig. 2B).

The general pattern was that resistance rates in sewage isolates matched the resistance rates in clinical isolates such that the most common types and the least common types of resistance were the same in sewage and clinical isolates. A similar pattern could not be seen when gene-based sewage data was plotted against clinical resistance rates. For example, in all the ten included countries, ARGs conferring resistance to aminoglycosides was most common in sewage whereas it was the least common type of resistance in clinical isolates.

## 4 Discussion

To the best of our knowledge this is the first study comparing an isolate-based and a gene-based sewage surveillance approach in parallel by relating sewage data to the prevalence of resistance in clinical isolates. The evaluation of different models could not identify any of the approaches or one type of predictor being clearly superior across all investigated antibiotic classes. However, it provides some support that it, at least in certain cases, could be advantageous to combine isolate-based and gene-based sewage data for the assessment of resistance prevalence in clinical *E. coli* isolates.

In the present study, we have evaluated both antibiotic class-specific and non-specific sewage predictors, *i.e.* explanatory variables that mechanistically should be directly connected to resistance against a particular class of antibiotics and those that should not be. In univariable models, class non-specific sewage variables – such as MARI, *intI1* and total ARGs - correlated better with clinical resistance rates than class-specific sewage variables in some cases. Furthermore, for both gene-based data and isolate-based data the predictors showing the best mean fit in univariable models were non-specific (*intI1* and MARI, respectively). However, although class non-specific data could give information about the overall resistance landscape, it should still be desirable to build sewage surveillance systems on class-specific, or preferably antibiotic-specific, predictors. Not least since non-specific predictors have a limited chance to reflect a change in the local resistance landscape where an increase in resistance against one or some antibiotics is not accompanied by a proportionally similar increase in resistance against other antibiotics. Such changes could for example occur due to local antibiotic use patterns or the introduction of a successful bacterial clone with a specific resistance profile [33], [34]. Capturing sudden and/or dramatic increases in resistance to specific antibiotics is indeed one of the more important objectives of a surveillance system with the aim of guiding empirical antibiotic treatments.

Given the benefit of being able to assess resistance prevalence in clinical isolates based on antibiotic class/substance specific sewage predictors, as argued for above, the findings that such predictors were sometimes inferior to non-specific predictors warrant some reflection. When antibiotic class-specific, gene abundance predictors were used, it was evident that the fit was less good for the fluoroquinolone resistance models compared to the other models. This is likely because the most common determinants for fluoroquinolone resistance in *E. coli* are mutations in chromosomal genes encoding the targets of the antibiotics rather than mobile resistance genes. It could be possible to quantify such mutations by DNA sequencing, but it would require very deep metagenomic sequencing or sequencing of amplicons targeting quinolone resistance determining regions [35], [36]. While there are mobile fluoroquinolone resistance genes as well, which can also be detected in the sewage through metagenomic sequencing, they rarely increase minimal inhibitory concentrations for fluoroquinolones above clinical breakpoints on their own [37], [38]. Thus, the carriage of such genes is not necessarily indicative of bacteria classified as resistant. This could indeed be true for mobile resistance genes conferring decreased susceptibility to other classes of antibiotics as well [39], which also in those cases might limit possibilities to use the abundance of such mobile resistance genes as predictors of clinical resistance.

With regard to class-specific isolate-based predictors (resistance rates in sewage isolates), and as presented before [10], no correlation was seen with aminoglycoside or third-generation cephalosporin resistance in clinical isolates. There could be several possible reasons behind this. First, even if surveillance of resistance in many European countries is, from a global perspective, relatively comprehensive and standardized, the underlying data is in many cases far from perfect. Particularly important for introducing biases are differences in sampling strategies (*e.g.* from which patients isolates are collected for testing) and incomplete resistance profiling [20], [21]. Differences in both of these could lead to deceiving differences in clinical surveillance data between countries. Second, when analyzing sewage samples, any *E. coli* carried in the gut flora of people could likely be captured, not only those causing infections. If the gut *E. coli* do not reflect differences observed in clinical isolates between populations, neither would we expect sewage *E. coli* to do so. Third, reported differences between countries could be restricted to the hospitalized populations and not present or as pronounced between the general populations. This would result in a lack of correlation since hospitalized populations are the source for the clinical isolates whereas it is the general populations that should be best reflected by the municipal sewage samples included in the present study. Fourth, the relatively low prevalence of sewage *E. coli* isolates resistant to aminoglycosides and/or third-generation cephalosporins could lead to insufficient resolution in the data, preventing detection of existing differences (too low statistical power). The resolution could possibly be improved by a direct plating methodology where sewage samples are cultured on selective plates with and without antibiotic supplementation, followed by colony counting. However, when such an approach was used for assessing the relative abundance of third-generation cephalosporin resistance in sewage *E. coli*, differences reported for clinical isolates between countries could not be reflected [10].

Since no significant correlation was observed between resistance rates in sewage and clinical isolates for third-generation cephalosporins and aminoglycosides, it might appear surprising that sewage abundance of third-generation cephalosporin and aminoglycoside resistance genes most common in *E. coli* did show relatively strong relationships with corresponding resistance rates in clinical *E. coli*. This could give support for insufficient resolution when studying the prevalence of these types of resistances in sewage *E. coli* by the applied isolate-based methodology. On the other hand, none of the mobile ARGs most commonly found in *E. coli* are specific to this species. Therefore, it is notoriously difficult to link mobile ARGs to specific bacterial hosts, especially plasmid-borne ones, through metagenomic sequencing [40]. Hence, the genes detected by metagenomic sequencing could largely originate from non-*E. coli* bacteria harboring mobile ARGs identical or closely related to those that can be found in *E. coli*. In support of the latter is the finding that aminoglycoside resistance genes were among the most common classes of ARGs in sewage while aminoglycoside resistance is the rarest type of resistance observed in *E. coli* isolates (both sewage and clinical). In addition, analyses of fecal samples have shown that culture-based methodology could readily detect a specific third-generation cephalosporin resistance trait in *E. coli* (*bla*_CTX-M-15_) that in the great majority of cases remained undetected by metagenomic sequencing [41].

Although this study is the first to suggest a value of combining isolate-based and gene-based sewage data for assessing clinical resistance rates, the restriction to ten study sites/countries is a limitation. Studies including more study sites would allow more reliable validations of different models' predictive capacity. Countries/study sites selected for such endeavors should ideally cover various resistance landscapes and allow access to high-quality, representative clinical surveillance data, preferably from active surveillance efforts. Currently, we believe that the rarity of settings (countries) fulfilling this is one of the largest obstacles for benchmarking a sewage surveillance approach. The corresponding sewage data, whether metagenomic or isolate-based, is in comparison much easier to generate and less prone to sampling bias.

## 5 Conclusions

This study suggests a value of including several different types of resistance markers in sewage, and possibly also combining them, but also a need for further evaluations. When performing such evaluations, it is also important to reflect on that a surveillance system aiming at predicting resistance rates in clinical isolates should be able to capture changes in any type of resistance in defined species, regardless of whether it is accompanied by proportionally similar changes in resistance to other antibiotics or not. Consequently, if a sewage surveillance system should reach acceptance as a tool for guiding empirical antibiotic treatments, it most likely needs to rely on resistance markers in the sewage that are antibiotic (class) specific and can be linked to specific bacterial species. Measuring resistance rates in species-confirmed sewage isolates is an obvious way of generating such data and is also what would be most similar to the clinical resistance data used today for guiding empirical treatments. However, gene-based sewage surveillance relying on resistance markers that cannot be specifically linked to certain antibiotic classes or species could be more resource-efficient and still be valuable for assessing general trends between sites and over time.

## CRediT authorship contribution statement

**Carl-Fredrik Flach:** Writing – review & editing, Writing – original draft, Supervision, Resources, Project administration, Methodology, Investigation, Funding acquisition, Conceptualization. **Fanny Berglund:** Writing – review & editing, Visualization, Software, Methodology, Formal analysis. **Gilbert Osena:** Writing – review & editing, Visualization, Software, Methodology, Formal analysis. **Patricia M.C. Huijbers:** Writing – review & editing, Resources, Methodology. **D.G. Joakim Larsson:** Writing – review & editing, Resources, Conceptualization.

## Funding

This research was funded by the 10.13039/100010269Wellcome Trust [222897/Z/21/Z], the 10.13039/501100001862Swedish Research Council Formas [2021-00922], and the Region Västra Götaland under the ALF agreement [ALFGBG-978722 and ALFGBG-1005684]. For the purpose of open access, the authors have applied a CC BY public copyright license to any Author Accepted Manuscript version arising from this submission.

## Declaration of competing interest

The authors declare that they have no known competing financial interests or personal relationships that could have appeared to influence the work reported in this paper.

## Data Availability

Data will be made available on request. The raw DNA sequencing data has been submitted to the European Nucleotide Archive and connected to the Project PRJEB87859. All R scripts are publicly available at GitHub: https://github.com/gilbertella/semar_wp1.
