# Supplementary material for: Sewage surveillance for assessing clinical antibiotic resistance prevalence: Combining metagenomic and phenotypic data
Source: One Health. 2026 Jun 18;23:101485. doi: 10.1016/j.onehlt.2026.101485 (PMC13316215; doi:10.1016/j.onehlt.2026.101485)
Supplement: Supplementary file 1 [file mmc1.docx]

**Table S1.** Data on number of clinical invasive E. coli isolates tested and prevalence of resistance against four classes of antibiotics in ten European countries obtained from the 2016 and 2017 EARS-Net reports

| **Country** | **Year**^1^ | **AP** | | **FQ** | | **3GC** | | **AG** | |
| --- | --- | --- | --- | --- | --- | --- | --- | --- | --- |
|  |  | **N** | **% R** | **N** | **% R** | **N** | **% R** | **N** | **% R** |
| Denmark | 2016 | 4885 | 45.6 | 5123 | 12.8 | 4883 | 6.9 | 5122 | 6.0 |
| Finland | 2016 | 2874 | 35.2 | 5305 | 12.0 | 5223 | 6.9 | 4982 | 5.0 |
| Norway | 2016 | 3731 | 42.2 | 3731 | 13.6 | 3734 | 5.9 | 3732 | 7.2 |
| Sweden | 2017 | 396^2^ | 34.1^2^ | 5762 | 15.8 | 5790 | 7.4 | 5758 | 6.5 |
| Belgium | 2017 | 4669 | 57.5 | 4382 | 23.8 | 4672 | 9.7 | 3769 | 8.1 |
| France | 2017 | 13293 | 55.6 | 13328 | 15.0 | 13352 | 10.2 | 13103 | 7.0 |
| Germany | 2017 | 19786 | 49.1 | 21080 | 20.9 | 21070 | 12.3 | 20623 | 6.9 |
| Greece | 2017 | 1306 | 57.5 | 1464 | 32.9 | 1470 | 18.3 | 1467 | 17.0 |
| Italy | 2017 | 4078 | 67.1 | 6945 | 44.9 | 7077 | 29.5 | 7134 | 18.4 |
| Spain | 2017 | 5724 | 62.6 | 5557 | 32.5 | 5804 | 12.8 | 5805 | 13.8 |

*AP: aminopenicillins; FQ: fluoroquinolones; 3GC: third-generation cephalosporins; AG: aminoglycosides; N: number of tested isolates; % R: percent of isolates resistant to the tested class of antibiotics*

^1^*Data was obtained from either the 2016 or the 2017 EARS-Net report to match the year when the analyzed sewage sample was collected in each country.*

^2^*In Sweden, testing with ampicillin was replaced by testing with amoxicillin–clavulanic acid in 2016, therefore the data presented are from 2015.*

**Table S2.** Gene clusters included in Top ten *E. coli* ARG variables

| **Top ten E. coli ARGs** | **Top ten E. coli AP ARGs** | **Top ten E. coli FQ ARGs** | **Top ten E. coli 3GC ARGs** | **Top ten E. coli AG ARGs** |
| --- | --- | --- | --- | --- |
| *aadA22* | *bla*_CMY-129_ | *oqxA* | *bla*_CMY-129_ | *aac(3)-IIc* |
| *aph(3’’)-Ib* | *bla*_CTX-M-186_ | *oqxB* | *bla*_CTX-M-186_ | *aac(3)-IVa* |
| *aph(6)-Id* | *bla*_CTX-M-21_ | *qepA1* | *bla*_CTX-M-21_ | *aac(3)-VIa* |
| *bla*_CTX-M-186_ | *bla*_HERA-5_ | *qepA3* | *bla*_DHA-17_ | *aadA22* |
| *bla*_TEM-115_ | *bla*_NDM-9_ | *qnrA3* | *bla*_KPC-12_ | *aadA2b* |
| *mph*(A) | *bla*_OXA-16_ | *qnrB65* | *bla*_KPC-9_ | *aadA5* |
| *sul1* | *bla*_OXA-514_ | *qnrD2* | *bla*_NDM-9_ | *aph(3’)-Ia* |
| sul2 | *bla*_OXA-534_ | *qnrS3* | *bla*_OXA-16_ | *aph(3’’)-Ib* |
| *tet*(A) | *bla*_SHV-78_ | *qnrS6* | *bla*_SHV-78_ | *aph(4)-Ia* |
| *tet*(B) | *bla*_TEM-115_ | *qnrVC7* | *bla*_TEM-115_ | *aph(6)-Id* |

**Table S3.** Obtained adjusted R^2^ values from beta regression analysis, clinical resistance rates vs. sewage data

|  | **AP-res** | **FQ-res** | **3GC-res** | **AG-res** |
| --- | --- | --- | --- | --- |
| **Gene-based data** |  |  |  |  |
| Total ARGs | 0.41 | 0.38 | 0.37 | 0.54 |
| ARGs for respective antibiotic class | 0.65 | 0.29 | 0.56 | 0.73 |
| Top ten *E. coli* ARGs | 0.55 | 0.65 | 0.50 | 0.74 |
| Top ten *E. coli* ARGs for respective antibiotic class | 0.67 | 0.29 | 0.72 | 0.74 |
| *intI1* | 0.57 | 0.70 | 0.53 | 0.74 |
| **Phenotypic isolate-based data** |  |  |  |  |
| Resistance rates for respective antibiotic class | 0.62 | 0.50 | -0.12 | 0.04 |
| MARI | 0.55 | 0.44 | 0.25 | 0.43 |
| **Gene-based + isolate-based data** |  |  |  |  |
| *intI1* + resistance rates | 0.73 | 0.66 | 0.47 | 0.71 |
| *intI1* + MARI | 0.62 | 0.68 | 0.47 | 0.71 |
| Top ten *E. coli* ARGs + resistance rates | 0.76 | 0.63 | 0.44 | 0.70 |
| Top ten *E. coli* ARGs + MARI | 0.66 | 0.67 | 0.45 | 0.75 |
| Top ten *E. coli* ARGs for respective antibiotic class + resistance rates | 0.82 | 0.62 | 0.71 | 0.70 |
| Top ten *E. coli* ARGs for respective antibiotic class + MARI | 0.75 | 0.68 | 0.69 | 0.76 |

**Table S4.** Obtained mean absolute percentage errors (MAPE) from leave-one-out cross-validations

|  | **AP-res** | **FQ-res** | **3GC-res** | **AG-res** |
| --- | --- | --- | --- | --- |
| **Gene-based data** |  |  |  |  |
| Total ARGs | 16.5 | 44.0 | 39.8 | 33.4 |
| ARGs for respective antibiotic class | 12.8 | 42.1 | 36.3 | 26.0 |
| Top ten *E. coli* ARGs | 14.4 | 28.0 | 32.9 | 21.2 |
| Top ten *E. coli* ARGs for respective antibiotic class | 11.8 | 42.3 | 27.4 | 22.2 |
| *intI1* | 14.2 | 23.8 | 30.3 | 19.8 |
| **Phenotypic isolate-based data** |  |  |  |  |
| Resistance rates for respective antibiotic class | 14.1 | 43.0 | 59.2 | 44.4 |
| MARI | 15.0 | 45.9 | 73.1 | 46.3 |
| **Gene-based + isolate-based data** |  |  |  |  |
| *intI1* + resistance rates | 11.4 | 35.1 | 36.5 | 20.9 |
| *intI1* + MARI | 15.1 | 32.3 | 58.1 | 32.3 |
| Top ten *E. coli* ARGs + resistance rates | 12.2 | 40.1 | 36.7 | 24.0 |
| Top ten *E. coli* ARGs + MARI | 15.2 | 35.5 | 60.5 | 28.0 |
| Top ten *E. coli* ARGs for respective antibiotic class + resistance rates | 10.8 | 36.8 | 30.5 | 24.0 |
| Top ten *E. coli* ARGs for respective antibiotic class + MARI | 13.0 | 30.8 | 47.0 | 27.1 |


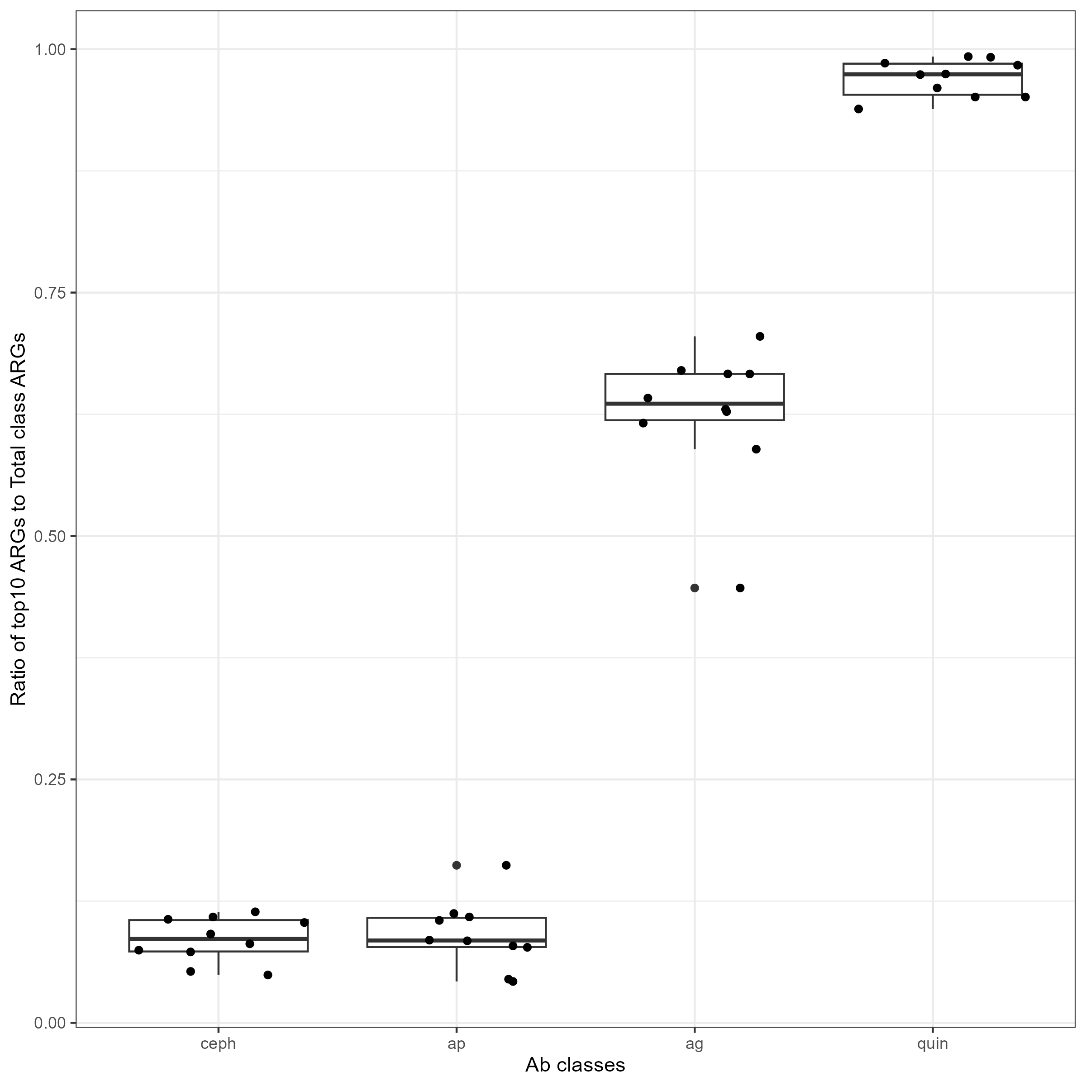


**Fig S1.** Abundance proportion of top ten ARGs for the third generation cephalosporin (3GC), aminopenicillin (AP), aminoglycoside (AG) and fluoroquinolone (FQ) antibiotic classes in relation to the abundance of total ARGs for the corresponding class. Each datapoint represents a country/sewage sample. The boxes represent the 25^th^ and 75^th^ percentiles, the whiskers show the highest and lowest values, excluding the outliers, and the horizontal lines represent the medians.

1. Aminopenicillin resistance models


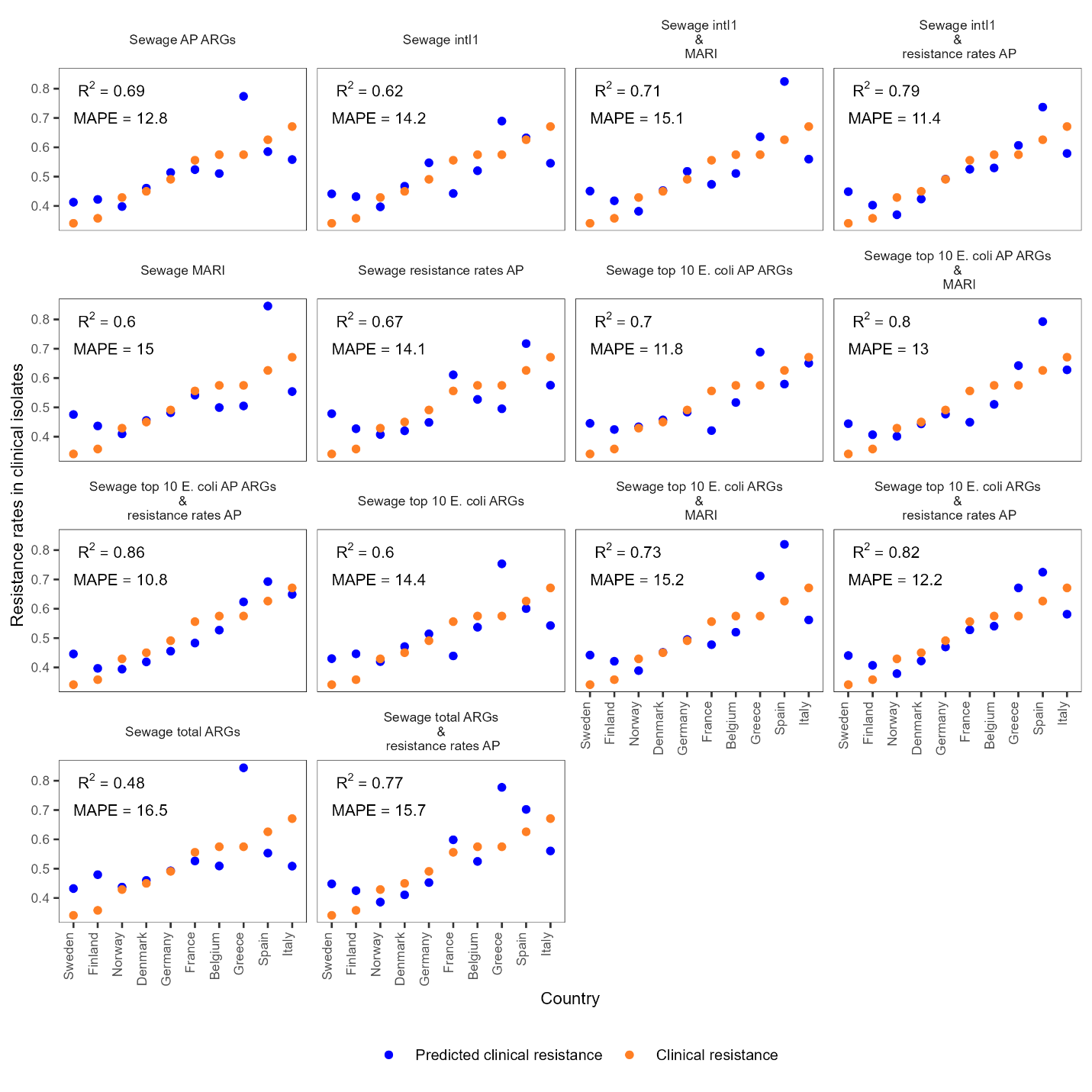


1. Fluoroquinolone resistance models


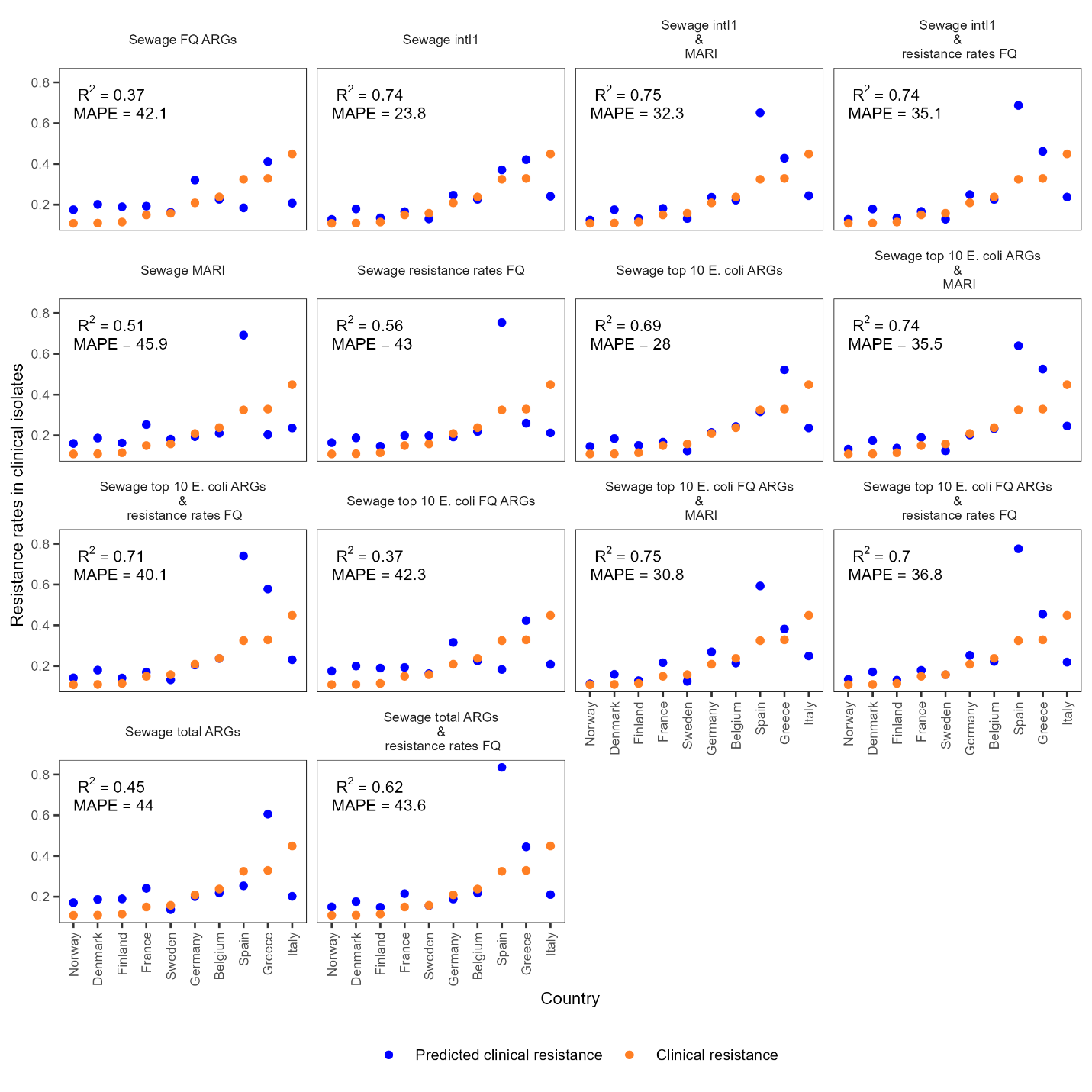


1. Third generation cephalosporin resistance models


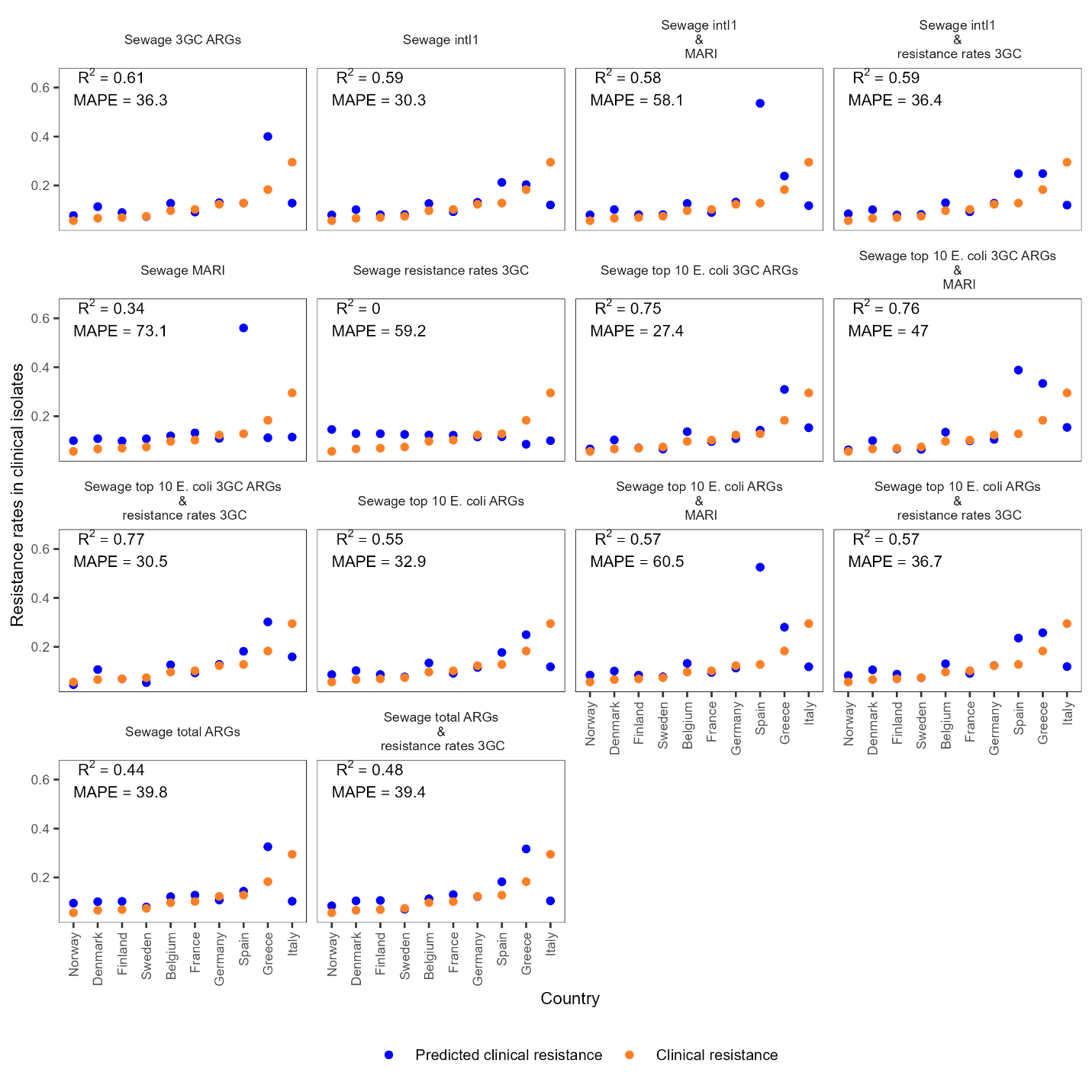


1. Aminoglycoside resistance models


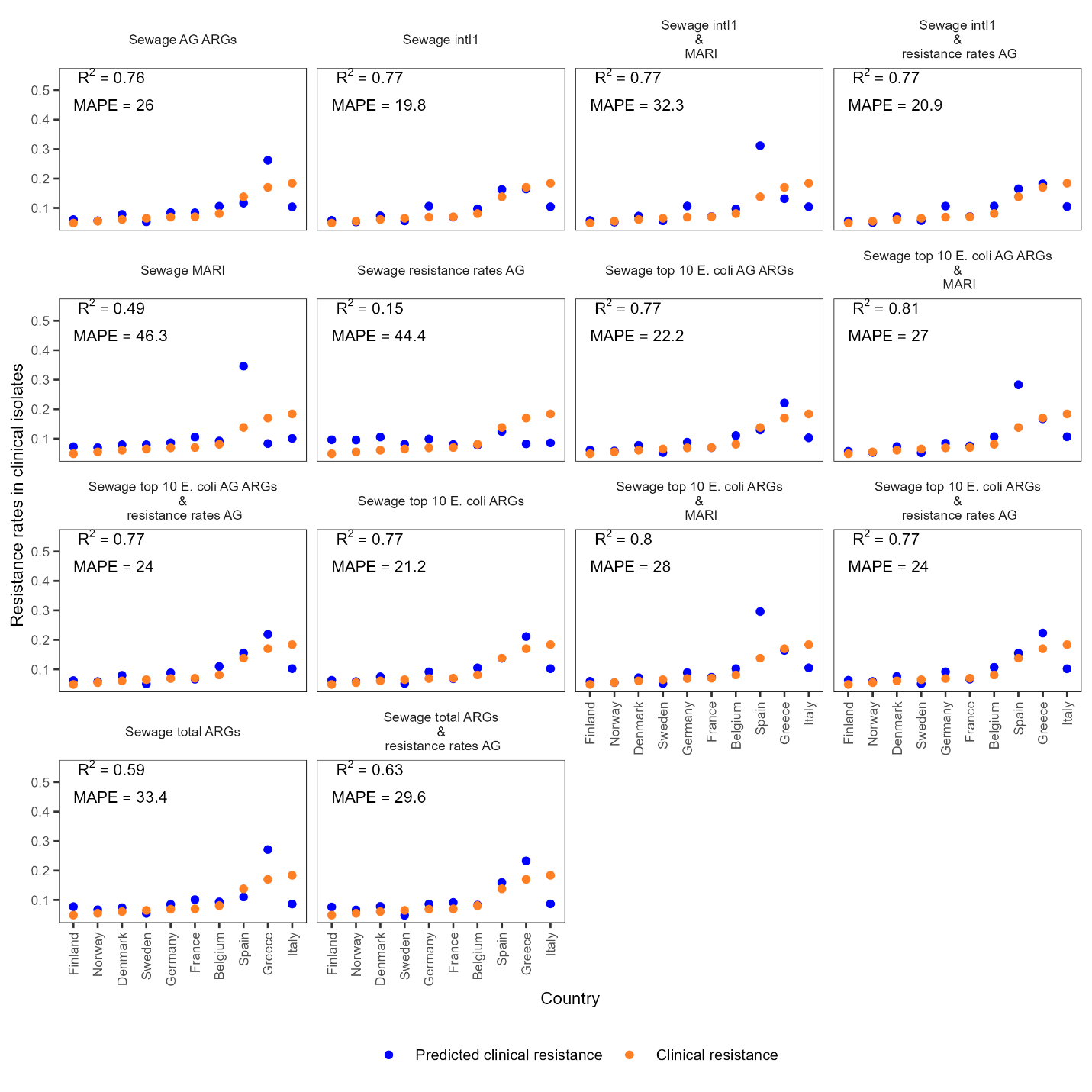


**Fig S2.** Predictions of clinical resistance prevalence from leave-one-out cross-validations of beta regression models based on sewage phenotypic and/or genomic resistance data. The orange dots represent reported clinical resistance while the blue dots the predicted values from each beta regression model. The predictor variables used in each model are indicated at the top of each subplot. Pseudo R2 from each model and mean absolute percentage error (MAPE) are shown on each subplot. The four panels (A-D) represent the aminopenicillin (AP), fluoroquinolone (FQ), third-generation cephalosporin (3GC), and aminoglycoside (AG) resistance models, respectively.
